# Cohort profile: Design and methods for Project HERCULES (Healthcare Exemplar for Recovery from COVID 19 Using Linear Examination Systems): Multi-disciplinary implementation and evaluation of an asynchronous review clinic in NHS eye-care services

**DOI:** 10.1371/journal.pone.0330863

**Published:** 2025-09-10

**Authors:** Dhakshi Muhundhakumar, Caroline S. Clarke, Grant Mills, Angus I. G. Ramsay, Kerstin Sailer, Peter Scully, Duncan Wilson, Dun Jack Fu, Siyabonga Ndwandwe, Rosica Pachilova, Anne Symons, Steve Napier, Joy Adesanya, Gus Gazzard, Robin Hamilton, Jonathan Wilson, Paul Webster, Peng Tee Khaw, Sobha Sivaprasad, Hari Jayaram, Paul J. Foster, on behalf of the HERCULES Consortium

**Affiliations:** 1 NIHR Biomedical Research Centre at Moorfields Eye Hospital NHS Foundation Trust and Institute of Ophthalmology University College London, London, United Kingdom; 2 Research Department of Primary Care and Population Health, University College London, London, United Kingdom; 3 Bartlett Faculty of the Built Environment, University College London, London, United Kingdom; 4 Research Department of Behavioural Science and Health, University College London, London, United Kingdom; 5 Patient Representative, Edinburgh, United Kingdom; 6 Moorfields Eye Hospital NHS Foundation Trust, London, United Kingdom; 7 Operations and Product Management, Ubisense Ltd, Cambridge, United Kingdom; West Bengal University of Animal and Fishery Sciences, INDIA

## Abstract

**Objectives:**

To describe the research principles and cohort characteristics of the multi-disciplinary Project HERCULES, an innovative model of safe high-volume outpatient eye-care service for patients with stable chronic eye diseases. Results and analyses of the workstreams within Project HERCULES will be reported elsewhere. The rationale was to improve eye-care capacity in the National Health Service (NHS) in England through the creation of technician-delivered monitoring in a large retail-unit in a London shopping-centre, with remote asynchronous review of results by clinicians (named Eye-Testing and Review through Asynchronous Clinic (Eye-TRAC)). UCL’s Bartlett School of Sustainable Construction developed the RIBA (Royal Institute of British Architects) Stage 1 briefing requirements for optimal design specifications for this model of care from first principles research, by analysing ergonomic data from multiple iterations.

**Methods:**

Patients aged 18 years or above being monitored in secondary care in Moorfields Eye Hospital NHS Trust for stable glaucoma or retinal conditions were given appointments at Eye-TRAC at Brent Cross, London. Patients were also recruited at City Road and Hoxton Eye-TRACs, as comparators for the motion tracking study. Willing participants were recruited when attending Eye-TRAC from 11^th^ October 2021–1^st^ December 2023, during this time four spatial “iterations,” with different configurations of equipment were investigated in succession. Recruited participants provided information on their eye health and quality of life via a questionnaire, as well as agreeing to wear a tracking device. The tracking device was worn for the duration of their clinic visit and along with directly observed timings built a picture of patient and staff flow through the clinic. Rapid qualitative and ethnographic analyses were conducted, drawing on staff, manager and patient interviews and observation of service delivery and challenges. Separately, anonymised data from across the Trust informed an analysis of the impact of opening the Eye-TRACs on Trust-wide waiting times. A nationwide discrete choice experiment was also conducted to assess patients’, healthcare providers’, and the public’s preferences for key service features..

**Findings to date and conclusion:**

41,567 patients attended the Brent Cross Eye-TRAC between 11^th^ October 2021 and 1^st^ December 2023. 5,539 patients were recruited to Project HERCULES. Spatial configurations promoting independently parallel patient journeys with limited queuing, and direct line of sight between diagnostic stations, supported efficient patient flow. The latter iteration incorporated cataract clinics. Although it added more system complexity, it enabled the evaluation of a further indication for use of Eye-TRAC.

**Future plans:**

The analysis of trust-wide data on the impact of the Eye-TRACs on waiting times and a nationwide evaluation of stakeholders’ preferences regarding diagnostic and monitoring services for stable disease are underway. We will also identify and enumerate limitations in information technology that create bottlenecks in the review process. Qualitative analysis of patient and staff feedback alongside rapid ethnographic work to streamline services is also under way. We seek to develop a framework to help inform NHS guidance and future service planning for ophthalmology and other outpatient diagnostic services. Our data will be analysed to identify enhancements to further streamline operational efficiency.

## Introduction

### Impact of COVID-19 on outpatient ophthalmology services

The COVID-19 pandemic precipitated the greatest health crisis in living memory [1]. The suspension of non-urgent NHS work led to millions more delayed appointments in an already strained eyecare service across the United Kingdom and, after years of health funding austerity, this backlog was potentially insurmountable. Overall, NHS England surgical waiting lists rose from 4.4 million pre-pandemic to over 8 million (December 2023) and have been projected by the National Audit Office to rise to 12 million by 2025 [2]. Ophthalmology, as the busiest outpatient specialty (with over 8 million annual attendances in the NHS in England [3]) was severely affected. Before the pandemic (2017), 3,384 ophthalmology patients in the UK suffered follow-up delays greater than a year, with 1% of these suffering preventable loss of vision [4]. In 2020, the Health Services Safety Investigations Body published a report on lack of timely glaucoma monitoring and inadequate hospital eye service capacity after an incident where a 34-year-old woman became blind, and highlighted the need for hospital eye services to work innovatively to deliver timely care [5]. Following the first lockdowns of the pandemic in March 2020, over one million NHS ophthalmology appointments were deferred, adding to the huge existing backlog and the potential for thousands of cases of avoidable blindness [5].

In 2013, direct costs of NHS eye care were £3 billion in the UK, with a further £6 billion/year of indirect costs of sight loss [6]. People fear blindness more than severe angina or kidney dialysis [7]. Patients suffering from chronic eye disease comprised a high-risk demographic for COVID-19 morbidity and mortality, most being elderly, and many with multiple systemic co-morbidities and disproportionately of minority ethnic heritage [8,9].

### Rationale for a novel approach to monitoring chronic eye diseases

Glaucoma, age-related macular degeneration (AMD), diabetic retinopathy (DR) and cataract are increasing in prevalence due to an ageing population, and are being identified and referred earlier due to the availability of advanced imaging technologies such as optical coherence tomography (OCT) in high street optometry practices [10]. These patients need regular imaging and functional testing to monitor their chronic and often lifelong eye conditions [11].

Under traditional care pathways, patients with stable glaucoma, AMD and DR attend outpatient appointments in secondary care. These appointments can take several hours, with large numbers of patients sitting in waiting rooms that are frequently cramped and poorly ventilated. During the appointment, they may interact with nurses, technicians, optometrists and doctors, with a waiting time between seeing each health professional and the various tests required. In view of the increasing capacity constraints, the dangers of COVID-19 and other air-borne disease transmission, and the vulnerable population that attend these clinics, it has become increasingly accepted that the traditional outpatient model of delivering care for chronic eye disease needs to be re-appraised [12,13].

During the inception of Project HERCULES in April 2020, minimising the potential for COVID-19 transmission was the overriding priority. Since then, the focus has shifted to building more high-volume chronic eye disease monitoring capacity in the form of diagnostic and monitoring clinics to overcome backlogs and increase patient throughput within NHS services [14] including, more recently, cataract surgery.

In the context of significant capacity challenges, more efficient, higher volume services are needed to monitor chronic stable patients and identify those in need of treatment escalation [15]. An adjunct to increased capacity is risk stratification, based upon relevant data, allowing the identification of higher risk patients who are more susceptible to harm from delayed appointments [16].

Asynchronous review, or “virtual” clinics (as they have been previously broadly referred to) are well established as a model of care [17–27] whereby patients undergo testing by technicians with results being reviewed by clinicians at a later time. Moorfields established “virtual diagnostic and monitoring clinics” in the main hospital in 2014 (Clinic 1A) [28], and thereafter expanded the service to other sites. They have become part of the “new normal” landscape of hospital ophthalmology services [13,17–19,29,30], providing a route to increasing clinic capacity in the face of otherwise insurmountable backlogs [21,22,31,32] for patients whose clinical condition is deemed “probably stable” [28]. These clinics are led by ophthalmic technicians, with operational protocols to examine patients in a fraction of the time of a conventional hospital appointment, thus reducing staffing costs and increasing productivity. Test results are reviewed asynchronously and remotely by specialist clinicians [29,33] potentially enabling them to assess a larger number of patients per session when compared to the conventional face-to-face model. We refer to these as Eye-Testing and Review through Asynchronous Clinics (Eye-TRACs) in this paper.

### Aims and objectives

This project aimed to provide research evidence on how to build additional clinic capacity focussing on patients requiring monitoring for chronic eye diseases such as AMD, DR and glaucoma, and latterly added cataract services.

Within this reconfigurable laboratory of clinical innovation, we gathered data on:

- Patient and staff movement through an examination pathway, using a combination of direct observation, real-time positional sensor tracking, equipment time stamps and administrative records of arrival and departure times, to identify and overcome capacity bottlenecks.
- How reconfiguration of the physical layout of the clinic influenced efficiency, performance and patient experience.
- The operational impact of introducing pre- and post-operative cataract services to the Eye-TRACs.

In parallel, we are reviewing the features associated with greater efficiency in existing high-volume cataract services in the UK and globally.

Through analysis of quantitative datasets and qualitative feedback, we aimed to formulate an evidence-based toolkit to reproduce high-efficiency and high-satisfaction monitoring pathways for chronic conditions within global healthcare systems.

## Methods

### Ethics approval

Project HERCULES was approved by North East – York NHS Research Ethics Committee (IRAS 303760). Ethical approvals were obtained prior to commencing recruitment and data collection. Participants were recruited to the trial after completing a written consent form.

### Study design and setting

The HERCULES project comprised the work of many different teams; these workstreams are summarised in Table 1. The HERCULES Eye-TRACs (described below) were used for the motion studies, patient reported outcome measures (PROMs) questionnaires and qualitative work. The discrete choice experiment (DCE) and ITSA (interrupted time series analysis) methods are described in the Quantitative analyses section.

**Table 1:** Workstreams within the HERCULES project.

WORKSTREAM *(& cohort groups involved)*	KEY RESEARCH QUESTIONS	METHODOLOGIES	DATA COLLECTED	OUTPUTS
**Motion tracking (Ubisense)** *Staff and patients at Brent Cross Eye-TRAC*	How do patients and staff move through the Eye-TRAC and how does layout affect flow and efficiency?	Ultra-wideband real-time location tracking (Ubisense,) spatial layout analysis	Movement trajectories, positional mapping	Comparisons between clinic iterations and layouts; identifications of bottlenecks
**Directly Observed Timings (DOTS)** *Staff and patients at Brent Cross, Hoxton, City Road Eye-TRAC*	What is the duration of diagnostic tests and total visit time across iterations and comparator clinics?	Manual observation and timing by UCL Bartlett researchers, architectural layout analysis: spatial syntax and queuing simulations	Task durations and total journey time	Comparison across design iterations and clinic types, architectural layout and spatial syntax analysis
**Time-stamped equipment logs and attendance records** *Brent Cross Eye-TRAC*	What is the patient visit duration and how long is spent on each piece of equipment? Is this impacted by disease severity?	Extraction of test durations from equipment and review of patient attendance logs; stratification of glaucoma patient visual field (VF) loss	Test durations, visit length, VF scores	Comparison across design iterations and clinic types, impact on patients based on severity of VF loss
**Clinic Spatial Design/ Simulation** *Brent Cross, Hoxton Eye-TRAC*	Which spatial layouts best support throughput, efficiency and safety?	Iterative redesign with simulation and real-time testing	Layout schematics, process observations	Performance across iterations
**Patient Reported Outcome Measures (PROMS)** *Eye-TRACs and face to face clinics*	How do patients perceive visual function, quality of life and service experience?	Questionnaires (VF-14, EQ-5D-5L with vision bolt-on,) content analysis of free-text responses.	PROM scores, free-text responses	Descriptive summaries, future mapping to utility scores
**Rapid qualitative work and Ethnography** *Staff and patients at EYE-TRACs and face to face clinics*	How were services designed and put into action? What were staff and patient experiences of this?	Review of clinic operational procedures, clinic observations, patient, staff and manager interviews	Field notes, observations, interview responses	Service implementation and challenges, staff adaptations and patient experience
**Interrupted Time Series Analysis (ITSA)** *Moorfields wide appointments*	Has the Eye-TRAC model reduced appointment delays across Moorfields sites?	Based on the latest clinically appropriate appointment date (LCAD) and actual appointment date, appointment delays were calculated	Review of Moorfields appointment records from 2018-2023	Comparison of appointment delays before and after the introduction of HERCULES Eye-TRAC
**Discrete Choice Experiment (DCE)** *UK wide national survey*	What are stakeholder preferences for different service attributes?	National survey quantifying trade-offs stakeholders are willing to make when accessing outpatient diagnostic services for chronic eye disease	Participant responses to hypothetical clinic scenarios	Based on responses, calculation of relative importance of various service features

#### Project HERCULES Clinic – A reconfigurable clinical innovation laboratory.

A retail unit in a major north London shopping mall (Brent Cross) was identified as a suitable site for an Eye-TRAC. Building on prior research and innovation [17–19,21–27,31], the Eye-TRAC was built in this rapidly converted retail space.

This new high throughput Eye-TRAC commenced with a linear flow design, followed by three further rapid iterations of physical layout and processes. This was enabled by a reconfigurable partition system developed by the Bartlett School of Architecture which used Design for Manufacture principles (minimize the number of parts, use standardised parts, create modular designs, use sustainable components with short, wide supply chains) to incorporate innovations, such as clustered or zonal use of diagnostic equipment. We intended also to examine how we might improve efficiency through optimisation of the physical layout of the clinic while minimising the risk of aerosol transmission to vulnerable patients [34]. The study commenced on 11th October 2021 and closed recruitment on 1st December 2023.

### Alternative comparator clinics

Moorfields Eye-TRACs at Hoxton, Cayton Street and City Road were used as comparator clinics for direct observed timing (DOTs) of patient journeys, patient reported outcome measures (PROMs) questionnaires and qualitative work (interviews of patients and staff). These are pre-existing asynchronous review clinics for stable glaucoma and retina patients – they were previously known as “virtual clinics” but for consistency of terminology will be referred to as Eye-TRACs. These clinics represent standard asynchronous models within Moorfields and provide a benchmark for evaluating the novel Brent Cross model.

Traditional hospital clinic outpatient care (face to face clinics) at Moorfields City Road site acted as an additional comparator. This was the commonest consultation type (~80%) before the pandemic.

### Cohort selection, recruitment and assessment

Table 1 summarises the different analyses carried out. The cohort recruited at the Eye-TRACs are summarised below; the data from these patients were used in the movement studies, PROMS and qualitative work. Separate cohorts were used for the nationwide discrete choice experiment (DCE) (covering a UK wide cohort of patients and staff) and interrupted time series analysis (ITSA) (reviewing Moorfields appointment delays over between 2018–2023.)

Eligible patients were those attending glaucoma and retina Eye-TRACs or latterly cataract clinics over the age of 18 and living locally. Prior to arrival at the clinic, all eligible patients were sent information on our research. They were advised that they may be asked if they wished to participate in the research project, which would involve wearing a tracking device during their visit and completing patient-reported outcome measure (PROM) questionnaires including demographic, visual status and quality of life (QOL) information and other patient reported outcome measures (see appendix) at the end of their appointment, and they were free to decline with no alteration in the care they would receive. On arrival, eligible patients were offered the opportunity to participate in the research. Not all eligible patients were approached, as it depended on the availability of members of the research team.

All patients then passed through the clinic, starting their journey with a health status review by an ophthalmic technician. This was followed by assessments of visual acuity, visual fields, intraocular pressure, OCT imaging and widefield fundus photography. Cataract and retina patients underwent pharmacological pupil dilation following initial visual acuity and intraocular pressure assessment. The diagnostic equipment included Humphrey Field Analysers 3 (HFA3) and optical coherence tomography (OCT) devices (Cirrus OCT 6000, Zeiss, Germany, for glaucoma; Spectralis OCT, Heidelberg Engineering, Germany, for retina; and ultra-widefield retinal imaging devices from Optos, Dunfermline, UK). Following asynchronous clinical review, clinicians could call patients to discuss any escalation in treatment (and contact pharmacy to post out any new drop treatment) and liaise with the administration team via the electronic patient record to organise appropriate follow ups.

### Measures

The study focussed on the following priority areas within the Eye-TRACs:

#### Monitoring of patient and staff movement.

We aimed to capture directly-observed, real-time measures of test duration and overall patient journey across existing similar Moorfields clinics, and four different experimental iterations of the Eye-TRACs, in a subset of participants at each site. This was performed by participant observers from the UCL Bartlett School of Architecture. Additionally, at the HERCULES Eye-TRAC patient and staff movements within the clinical facility were tracked using ultra-wideband real-time location systems (Ubisense, Cambridge, UK) during the four iterative configurations of the diagnostic clinic. Patients and staff gave written informed consent for this monitoring. Patients also gave written consent to access to their basic relevant clinical data and were asked for consent to be contacted to provide feedback on patient panels and participate in future related projects.

#### Implementation, layout efficiency, service performance, patient and staff experience.

Researchers from the UCL Research Department of Behavioural Science and Health used qualitative approaches (stakeholder interviews and non-participant observations) to study implementation of the new service and how staff and patients experienced it. They carried out a rapid ethnographic study to explore adverse experiences of the service, such as excessively long appointments and missed diagnostic tests. They carried out semi-structured interviews with staff, attended briefing meetings before shifts and observed (with written consent) staff-patient interactions, to develop guidance on how to avoid unintended outcomes.

The UCL Bartlett School of Sustainable Construction and UCL Bartlett Centre for Advanced Spatial Analysis investigated the features of the various clinic layouts that worked well and those that did not. This involved generating testable clinic design ideas, pre-testing the evidence through simulation, building prototypes, analysing the results using the data generated by the ultra-wideband real-time location system and sharing learning for redesigning. This work demonstrated the principles of building physical/ digital twin clinics which allow the development of a “living lab” that can set the standard for a novel experimental approach to optimising layout and building designs for healthcare.

#### Quantitative analyses of the healthcare system beyond Brent Cross.

Health economists from the UCL Research Department of Primary Care and Population Health are using interrupted time series analysis (ITSA) of anonymised system-wide Moorfields patient-level data to evaluate the impact of the Brent Cross Eye-TRAC on appointment delays across the Moorfields network sites. They defined delays using the “latest clinically appropriate date” (LCAD) associated with each appointment across Moorfields network sites over time. The difference between the LCAD and the actual appointment date is used to quantify the delay in days between when a routine monitoring appointment should happen and when it did happen. This analysis uses appointment information recorded in Moorfields routine electronic health records from 2018 to 2023. Changes in the delays to routine appointments across all Moorfields patients in clinics including asynchronous and face-to-face models were compared in time periods before and after the HERCULES Eye-TRAC opened in October 2021. Another quantitative analysis is employing a Discrete Choice Experiment (DCE) survey to evaluate stakeholders’ preferences regarding provision of outpatient diagnostic services for stable eye disease. Respondents are asked to choose between pairs of hypothetical scenarios describing different clinic options, and their responses across a number of different pairs are analysed to allow calculation of the relative importance of each service feature, and quantify trade-offs stakeholders are willing to make (e.g., longer travel time for faster results). Full details for these analyses will be published separately and findings will be used to support NHS guidance on the design and scaling of diagnostic hubs like Eye-TRAC.

A further separate quantitative analysis will use the HERCULES patient-reported outcome measures captured from attendees at the Moorfields at Brent Cross Eye-TRAC to generate a mapping algorithm to transform condition-specific (the Visual Function Index, VF-14) to generic (EQ-5D-5L) QOL outcomes, for use in interpreting the condition-specific measure more broadly.

### Target populations and eligibility

Recruitment for the tracking study targeted adult patients (over 18 years of age) attending follow-up clinic appointments within the Cataract, Glaucoma and Medical Retina services at existing Moorfields sites (City Road and Hoxton Eye-TRACs) and the new Moorfields at Brent Cross Eye-TRACs. Staff working within these clinics were also recruited for the qualitative work.

For the Moorfields system-wide patient-level data for analysis of appointment delays, anonymised data from adult patients seen at Moorfields sites with stable glaucoma or retinal disease were used.

To assess stakeholder preferences at a national level for the DCE, surveys were sent to adult ophthalmology patients in the UK (over 18 years of age), health care professionals and members of the general public.

### Statistical analyses

**Fig 1:**
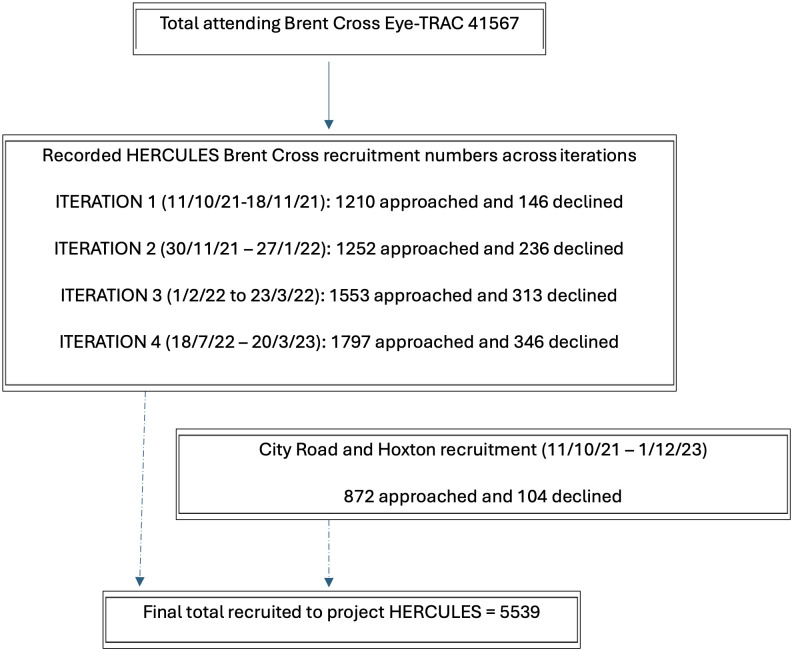
Flow chart showing numbers of patients approached by the recruitment team across iterations and numbers of patients declining participation.

This cohort profile primarily presents descriptive statistics to summarise patient demographics, diagnostic clinic attendance and service characteristics across the four design iterations. Details of patient numbers approached and declining participation in the study are presented as a flow chart (Fig 1). Detailed results from the motion tracking, ITSA, DCE, ethnographic and other analyses, including statistical techniques, will be presented in separate manuscripts.

### Data collection

#### Periods of recruitment: Follow up.

The Eye-TRACs at Brent Cross operated initially for four 8–12 week iterations seeing approximately 600 patients per week, with spatial and operational reconfiguration between each cycle. In between iterations patients continued to attend the Eye-TRACs and clinics ran as normal. Fig 1 demonstrates patients approached and declining participation in each iteration.

Some documented reasons for non-participation were:
“not interested” – 297 patients
“no English/ language barrier” – 144 patients
“no time” – 27 patients
“negative emotions” (upset or angry on the day) – 5 patients.

#### Quantitative data collection and analysis.

Participants who gave informed written consent at the start of their clinic visit were asked to complete the Visual Function Index (VF-14) [35] and the EQ-5D-5L [36,37] with vision “bolt-on” as part of the PROMs questionnaire.

Patient positional monitoring during the spatial reconfigurations provided the following data:

1. Examination duration and patient journey times through the ultra-wideband real-time location tracking system (Ubisense).
2. Directly observed patient journey times with contextual information with subsequent analysis of spatial viewsheds in the clinics from every location to every other location based on principles of space syntax [38], highlighting patient experience, flows and overlaps or spatial bottlenecks.
3. Time stamps recorded by ophthalmic equipment, and patient arrival and departure times recorded by clerical staff.

These data will be analysed to triangulate the “centre of gravity” of patient journey times, relating these to the directly observed contextual information, and to perform mathematical modelling of operational processes and patient flow.

#### Qualitative data collection and analysis.

At the end of the scheduled clinic attendance, patients were asked to complete a brief service user questionnaire as part of the same PROMs questionnaire that evaluated patient experience within the facility and how this compared to any prior diagnostic clinics they may have attended.

The PROMs questionnaires included both structured scales and optional free-text fields, with the latter being coded thematically (grouped into categories reflecting patients’ perceptions of clinic efficiency, communication, comfort and perceived safety) using qualitative content analysis techniques. These findings are being used to complement quantitative analyses and inform the ongoing refinement of patient-centred service design.

The Brent Cross experimental clinic ran for a 26-month period and our dataset will permit comparisons of patient experience across design iterations, across sites (Eye-TRACs at Brent Cross, Hoxton, City Road) and across clinic types (traditional “face to face” clinics and Eye-TRACs.)

The qualitative analysis of staff and patient interviews delivered both rapid, formative learning and summative lessons on planning, delivery and experience of this innovation.

Formative learning was provided via rapid qualitative analysis [39], operating in multiple cycles (reflecting the intervention being studied). Our data were drawn together using the Rapid Assessment Procedures (RAP) approach [40], which permits data collection and analysis to be conducted in parallel. The RAP sheets were updated after each instance of data collection (e.g., interview, meeting observation), facilitating quick and ongoing analysis and feedback with stakeholders.

A rapid ethnographic study employed semi-structured staff interviews, addressing the decision-making process in moving through the diagnostic pathway. As part of this study standard operating procedures were analysed and managers interviewed to give an understanding of expected service performance. Clinics and patients were then observed to identify unintended consequences (long appointments and missed diagnostic tests,) followed by technician interviews to understand how staff adapt to variability in patient needs and clinic flow.

The summative analysis will be organised around two broad themes, reflecting our research questions addressing implementation, delivery and experience of these services. Our analyses combined inductive theory (theory building) and deductive (theory-guided) approaches.

1. Service delivery and experience: The first analysis will address service delivery and experiences of the Eye-TRACs studied, from staff and patient perspectives. Key themes will include a) enabling patient access to service (e.g., service location and pre-appointment communication), b) organisation and delivery of testing (e.g., influence of different spatial layouts and how patients are taken round the service) and c) managing difficulties in service delivery (e.g., technical and informational).
2. Planning and implementation: The second analysis will focus on the approaches to designing and implementing service innovations. Key themes will include a) how different clinical, managerial and academic perspectives combined to address design and implementation issues, b) how engagement with frontline staff influenced approaches through different iterations and c) how learning from earlier iterations shaped approaches in later iterations.

#### Data management.

We have and will continue to adhere to institutional information governance policies which comply with the UK General Data Protection Regulation (GDPR) and Data Protection Act (2018) at all times so that personal identifiable information (PII) is protected.

Retrospective electronic patient records from the wider Moorfields dataset of all glaucoma and medical retina patients who have been seen at any site of the Moorfields Eye Hospital (MEH) NHS Foundation Trust between 2018 and 2023, provided by Dun Jack Fu with assistance from members of the Moorfields Service Improvement and Sustainability team, have been anonymised and transferred securely into the UCL Data Safe Haven, and are being used for an interrupted time series analysis to assess the impact of opening the Moorfields at Brent Cross Eye-TRAC on appointment delays. The Data Safe Haven is certified to the ISO27001 information security standard and conforms to NHS Digital’s Information Governance Toolkit.

Investigators at UCL were given data exports of the Brent Cross PROMs data containing only pseudonymised study data, following data quality checks by the Moorfields data management team. All study records will be retained for five years in secure storage within the Moorfields R&D offices following publication of the results.

#### Patient and public involvement and engagement (PPIE).

PPIE was incorporated in the iterative development of the Eye-TRACs and in developing and implementing the research methodology.

We are grateful to Ms Jocelyn Cammack and Mrs Helen Baker, at NIHR Biomedical Research Centre at Moorfields Eye Hospital NHS Foundation Trust & UCL Institute of Ophthalmology, London, UK, for their guidance on public and patient involvement. We have directly sought patients’ opinions for service development, throughout the iterative refinement of clinic design through qualitative surveys.

In terms of research and evaluation, one of the co-authors of this paper (SNa) is a PPIE collaborator and was fully involved in the quantitative, qualitative and rapid ethnography sections of this project. SNa attends regular project meetings, contributes to development of research materials, discussions of progress in the work, interpretation of findings and is a co-author of research outputs.

The project as a whole is supported by three major patient-centred charities that share its vision. These charities are Glaucoma UK, The Macular Society, and Diabetes UK. The charities were involved in the conception, design, and funding of the project and in disseminating findings to patient groups. They play a significant role in helping to develop patient and public involvement as this iterative project progresses.

## Results

41,567 patients attended the Moorfields Brent Cross Eye-TRAC between 11^th^ October 2021 and 1^st^ December 2023. 5,539 patients were recruited to Project HERCULES (which recruited from Eye-TRACs at Brent Cross, City Road and Hoxton.) This included 2,199 medical retina patients, 2,993 glaucoma patients, and 347 cataract patients. Mean age of the cohort was 64.6 years (standard deviation 13.3 years), minimum age was 18 years and maximum age was 98 years. Ethnicity profile reflects the diverse population of North London; 1444 patients were white British (26.1%), 1,208 patients were Indian (22%), 572 were black African or Caribbean (10.3%), 1232 patients (22%) did not declare their ethnicity.

Table 2 summarises the attendances for each specialty during and in between the iterations. The numbers recruited for HERCULES for each specialty is included in italics with an asterisk*.

**Table 2:** Patient numbers by service and date seen for NHS service at Moorfields Brent Cross and HERCULES recruitment (across sites).

Iteration	Service				Total
	Cataract	Glaucoma	Medical Retina	Support Services	
**Iteration 1** (11/10/21–18/11/21)					
Total patients		1019	607		1626
Recruited to HERCULES*		690*	348*		1038*
**Iteration 2** (30/11/21–27/1/22)					
Total patients		1380	850		2230
Recruited to HERCULES*		633*	472*		1105*
**Iteration 3** (1/2/22–23/3/22)					
Total patients		1511	1046		2557
Recruited to HERCULES*		885*	602*		1487*
**Iteration 4** (18/7/22–20/3/23)					
Total patients	1706	6798	5044	155	13703
Recruited to HERCULES*	347*	533*	615*		1495*
Other (11/10/21-1/12/23)					
(Total patients attending outside of the iteration dates)	1343	11475	8074	559	21451
Recruited to HERCULES*		252*	162*		414*
**Total** (11/10/21–1/12/23)	**3049**	**22183**	**15621**	**714**	**41567**
Total recruited to HERCULES*	***347***	***2993***	***2199***		***5539***

## Discussion

Iteration 1 tested a linear configuration, with patients and technicians completing tests with “lanes” of equipment, with a 1:1:1 ratio of equipment (e.g., for glaucoma 1 HFA for VF; 1 OCT; 1 widefield Optos). In iteration 2 a “clustered” model was introduced, with multiple devices of the same type placed in the same area, allowing technicians to quickly identify and utilise available machines rather than being restricted to the next one in a lane, where forward progress is dependent on the movement of the patient ahead. In iteration 3, an enhanced cluster design, the results of the qualitative work including rapid feedback exercises were utilised to try to increase efficiency gains.

Some of the rapid feedback exercises in iteration 4, examined the cause for excessively long appointments and found that complex patients who have significant additional care needs (either due to visual or other morbidity) were a major contributing factor to bottle-necks in the pathway. One interpretation is that to realise the potential efficiency gains of this method of working, dedicated diagnostic and monitoring clinics may be more suited, and more appropriately equipped, to deal with the larger number of mild/moderate disease severity cases, and that those with more complex disease, and systemic comorbidity, may be best cared for in a traditional hospital setting.

In iteration 4, “face to face” pre- and post-operative cataract clinics were introduced which created a more complex dynamic, and some disturbance in the smooth operating of the diagnostic and monitoring workstreams. We hypothesise that this was due to more complexity being introduced into the system and the loss of a consistent and predictable testing pathway that can occur when traditional face to face clinics are combined with Eye-TRACs. It demonstrates the need for design research into new models of care that involve various stakeholders in healthcare planning, architecture and new guidance development.

### Collaboration

The findings of this research project are of significant relevance to NHS eye care and other high-volume ambulatory outpatient services, in the post COVID-19 era. Research findings will be shared with the Royal College of Ophthalmologists, regional Integrated Care Systems and the NHS Department for Outpatient Transformation to help inform planning for future diagnostic clinic models across the United Kingdom.

Our research output has been and will be submitted for presentation at national and international conferences and for peer-reviewed publication in the domains of ophthalmology, patient experience, health economics, healthcare, built environment design and human-computer interaction.

The findings will be shared with the wider patient population in collaboration with our partners, the Macular Society, Glaucoma UK and Diabetes UK who are the leading patient-focused charities relating to eye-care in the United Kingdom.

Duncan Wilson’s team have provided motion tracking data on this public URL: https://github.com/djdunc/hercules/tree/main/data/live.

Kerstin Sailer’s team have provided data and protocols from the DOTS work stream on this public URL: https://datadryad.org/dataset/doi:10.5061/dryad.m0cfxppdj

All reasonable further data collaboration requests will be considered.

### Further details

In February 2024, when the rental contract for the original unit expired, the Eye-TRACs at Brent Cross transitioned to a new service facility at a neighbouring unit, serving medical retina, glaucoma, cataract and keratoconus patients. The new clinic aims to deliver at least 600 appointments per week.

### Future work

Longitudinal follow up of the HERCULES patient cohort is planned; to explore rates of treatment escalation and glaucoma progression in the patients seen in Eye-TRACs compared to face to face clinics.

#### High volume low complexity cataract services.

Iteration 4 of Project HERCULES saw the introduction of “one-stop” clinician-patient “face-to-face” cataract clinics, to serve new and post-operative patients and list patients for surgery. Cataract surgery is the most performed elective surgical procedure in the UK and demand for this procedure is ever-increasing with an ageing population and particularly after disruption to elective surgical lists during the pandemic. In a bid to improve the overall efficiency and accessibility of healthcare services, high-volume low-complexity (HVLC) surgical hubs have been proposed as a potential solution to the backlog of patients waiting for elective surgeries. The HERCULES group seeks to define the optimal design parameters for high-volume cataract surgical theatres in collaboration with researchers from the UCL Bartlett Faculty of the Built Environment using lessons learned during HERCULES so far.

A further extension to the project includes a rapid realist review (RRR) to explore the influence of the HVLC cataract surgery services on patient, service, and system outcomes, and how these interplay with implementation strategies, workforce dynamics, and the patient experience.

#### IT refinement.

Since the expansion of asynchronous “virtual” monitoring at Moorfields, we have found that clinical data review is one of the major challenges to efficiency in this care pathway. To address this, and to optimise processing efficiency and analysis of the large amount of clinical data and images acquired through the diagnostic clinic, researchers from the UCL Interaction Centre aim to create innovative strategies utilising artificial intelligence (AI)/ machine learning (ML).

This team will explore innovative solutions based on large language model processing and utilise voice recognition software to integrate clinician commentary on virtual reviews into the structured electronic medical record, with a secondary focus on ensuring the new system can provide data that is easily useable in future health services research.

## Conclusion

This manuscript describes the rationale for the various workstreams within the HERCULES project, a novel diagnostic model implemented to address capacity challenges in NHS eyecare services. The characteristics of the patients recruited at the HERCULES Eye-TRAC have been described and initial findings from the motion tracking studies described. This cohort profile lays the foundation for future analysis of service performance and patient and staff experiences. Results and findings will be presented in separate forthcoming manuscripts.

### Generalisability and application

We have demonstrated research and design principles associated with higher numbers of patients examined in our Eye-TRACs serving patients with chronic, presumed stable, eye disease, and believe these will be applicable to other high volume outpatient investigation and monitoring services beyond ophthalmology, both in the UK and globally. However, different cultural values in healthcare, and organisational environments, may create unforeseen operational variations or disruptions. Consequently, we are examining the impact of our clinic on waiting times across our NHS Trust’s network, and will work to build collaborations to further test our results and roll out this method of working across the UK and globally.

### Strengths and limitations of this study

- Our multi-disciplinary research team is a major strength of the work; new collaborations and understandings have arisen that cut across academic disciplines and we hope this will provide meaningful lessons for translating research into health service reform now and in future.
- Design development that used rapid experimentation to test new safe ideas (before spending significant resources on them) was employed; we collected data to build an evidence base, dynamically test new environments, build prototypes and execute analysis iteratively.
- Continuous input from technicians, administrative and managerial staff led to improvements in later iterations and greater buy-in from staff and ultimately the success of the project.
- Patient and public involvement was integral to the mixed-methods quantitative and qualitative research design.
- Due to the need for rapid service capacity expansion and high-volume throughput (as a result of the pandemic) the conditions and comparisons within the study could not be tightly controlled.

## Supporting information

S1 File HERCULES consortium list. A list of all members of the HERCULES consortium including their affiliations.
(PDF)
