# Supplementary material for: Cohort profile: Design and methods for Project HERCULES (Healthcare Exemplar for Recovery from COVID 19 Using Linear Examination Systems): Multi-disciplinary implementation and evaluation of an asynchronous review clinic in NHS eye-care services
Source: PLoS One. 2025 Sep 10;20(9):e0330863. doi: 10.1371/journal.pone.0330863 (PMC12422467; doi:10.1371/journal.pone.0330863)
Supplement: S1 File — A list of all members of the HERCULES consortium including their affiliations. (PDF) [file pone.0330863.s001.pdf]

Collaborators – Hercules Consortium

HERCULES Consortium: (FULL LIST with affiliations available as supplementary material)

Aadil Kazi – NIHR Biomedical Research Centre, Moorfields Eye Hospital & UCL

Angus Ramsay – Research Department of Behavioural Science and Health, UCL

Anne Symons – The Bartlett School of Architecture, UCL

Connor Beddow – Moorfields Eye Hospital NHS Foundation Trust

Chris Leak - Moorfields Eye Hospital NHS Foundation Trust

Caroline S Clarke – Research Department of Primary Care and Population Health, UCL

Dun Jack Fu - NIHR Biomedical Research Centre, Moorfields Eye Hospital & UCL

Duncan Wilson – Centre for Advanced Spatial Analysis, UCL

Dhakshi Muhundhakumar - NIHR Biomedical Research Centre, Moorfields Eye Hospital & UCL

Lina Song – School of Management, UCL

Declan Flanagan - Moorfields Eye Hospital NHS Foundation Trust

Elisha Chung - Moorfields Eye Hospital NHS Foundation Trust

Ella Preston - Moorfields Eye Hospital NHS Foundation Trust

Farbod Afshar Bakeshloo – Bartlett Faculty of the Built Environment, UCL

Giovanni Ometto – Faculty of Brain Sciences, UCL

Gus Gazzard - NIHR Biomedical Research Centre, Moorfields Eye Hospital & UCL

Grant Mills – The Bartlett School of Sustainable Construction, UCL

George Damianidis - Moorfields Eye Hospital NHS Foundation Trust

Helen Baker - Moorfields Eye Hospital NHS Foundation Trust

Hari Jayaram - NIHR Biomedical Research Centre, Moorfields Eye Hospital & UCL

Ian Eames – Department of Mechanical Engineering, UCL

Iqbal Fahmi – The Bartlett School of Sustainable Construction, UCL

Irinie Roufaeel – The Bartlett School of Sustainable Construction, UCL

Jocelyn Cammack - Moorfields Eye Hospital NHS Foundation Trust

Josefine Magnusson - Institute of Epidemiology and Health Care, UCL

Jemima Unwin – Bartlett School of Environment, Energy and Resources, UCL

Jonathan Wilson - Moorfields Eye Hospital NHS Foundation Trust

Joy Adesanya - Moorfields Eye Hospital NHS Foundation Trust

Kerstin Sailer – The Bartlett School of Architecture, UCL

Kathryn Scotcher - Moorfields Eye Hospital NHS Foundation Trust

Kimberley Quan - Moorfields Eye Hospital NHS Foundation Trust

Martin Utley – Clinical Operatinal Research Unit, UCL

Matala Dyke - Moorfields Eye Hospital NHS Foundation Trust

Naomi J Fulop – Department of Applied Health Research, UCL

Natalie O'Shea   - Moorfields Eye Hospital NHS Foundation Trust

Paul Foster – NIHR Biomedical Research Centre, Moorfields Eye Hospital & UCL

Peter Scully - Bartlett Faculty of the Built Environment, UCL

Paul Webster – Ubisense, Cambridge, UK

Paula Lorgelly – Department of Applied Health Research, UCL

Pei Li Ng – Department of Applied Health Research, UCL

Peter Thomas - NIHR Biomedical Research Centre, Moorfields Eye Hospital & UCL

Rachel Thompson - Moorfields Eye Hospital NHS Foundation Trust

Robin Hamilton - Moorfields Eye Hospital NHS Foundation Trust

Rosica Pachilova – Bartlett Faculty of the Built Environment, UCL

Rouba Ibrahim – School of Management, UCL

Susana Frazao Pinheiro – School of Management, UCL

Siyabonga Ndwandwe – Research Department of Primary Care and Population Health, UCL

Saheli Gandhi – The Health Care Organisation and Management Group, UCL

Samiul Alom - Moorfields Eye Hospital NHS Foundation Trust

Sherene Ettiene - Moorfields Eye Hospital NHS Foundation Trust

Sobha Sivaprasad – NIHR Biomedical Research Centre, Moorfields Eye Hospital & UCL

Stacey Angus - Moorfields Eye Hospital NHS Foundation Trust

Stephanie Kumpunen – Institute of Epidemiology and Health Care, UCL

Steve Napier – Patient Representative

Yue Tang – School of Management, UCL

Dominika Matusiak - The Bartlett School of Architecture, UCL

Ecem Ergin – The Bartlett School of Architecture, UCL

Xiaoming Li – Bartlett Faculty of the Built Environment, UCL

Muna Ayah - Moorfields Eye Hospital NHS Foundation Trust

Nadine Abdelgalil - Moorfields Eye Hospital NHS Foundation Trust

Paul Cartwright - Moorfields Eye Hospital NHS Foundation Trust

Sarah Davies - Moorfields Eye Hospital NHS Foundation Trust

Sandi Drewett - Moorfields Eye Hospital NHS Foundation Trust

Clare Feasby - Moorfields Eye Hospital NHS Foundation Trust

Simranjit Gill - Moorfields Eye Hospital NHS Foundation Trust

Steven Gill - Moorfields Eye Hospital NHS Foundation Trust

Nick Hardie - Moorfields Eye Hospital NHS Foundation Trust

Jamie Henderson - Moorfields Eye Hospital NHS Foundation Trust

Lesley Henry - Moorfields Eye Hospital NHS Foundation Trust

Peng Tee Khaw - Moorfields Eye Hospital NHS Foundation Trust

Richard Lee - Moorfields Eye Hospital NHS Foundation Trust

Sarah Martin - Moorfields Eye Hospital NHS Foundation Trust

Mary Masih - Moorfields Eye Hospital NHS Foundation Trust

Luke Nicholson - Moorfields Eye Hospital NHS Foundation Trust

Tulga Reis - Moorfields Eye Hospital NHS Foundation Trust

Nick Roberts - Moorfields Eye Hospital NHS Foundation Trust

Ana Sanchez - Moorfields Eye Hospital NHS Foundation Trust

Jon Spencer - Moorfields Eye Hospital NHS Foundation Trust

Karen Titmus - Moorfields Eye Hospital NHS Foundation Trust

Eleanor Dean – Akeso, London, UK

Nick Hynes – SOMO Global, London, UK

Olivia Jeffrey - Akeso, London, UK

Chris Robson - Akeso, London, UK

Tom Blair – Ubisense, Cambridge UK

Nick Burt – Institute of Ophthalmology, UCL

Dolores Conroy – Institute of Ophthalmology, UCL
